# Flower-like MoS_2_ microspheres compounded with irregular CdS pyramid heterojunctions: highly efficient and stable photocatalysts for hydrogen production from water

**DOI:** 10.1039/d1ra03834f

**Published:** 2021-06-30

**Authors:** Kai He, Liejin Guo

**Affiliations:** School of Mechanical Engineering, Shaanxi University of Technology Hanzhong 723000 China; International Research Centre for Renewable Energy, State Key Laboratory of Multiphase Flow in Power Engineering, Xi'an Jiaotong University Shaanxi 710049 China lj-guo@mail.xjtu.edu.cn +86 29 82669033 +86 29 82663895

## Abstract

An irregular CdS pyramid/flower-like MoS_2_ microsphere composite photocatalyst was successfully synthesized using a simple one-step hydrothermal method. The as-prepared samples were characterized by X-ray diffraction, X-ray photoelectron spectroscopy, scanning electron microscopy, ultraviolet visible absorption spectroscopy, fluorescence spectroscopy and photoelectrochemical tests. The composite photocatalysts showed superior photocatalytic activities for hydrogen evolution from water under visible light irradiation (*λ* ≥ 420 nm) with an extremely high apparent quantum yield (AQY = 64.8%) at 420 nm. To our knowledge, this value is the highest reported efficiency value for CdS/MoS_2_ photocatalysts. Further detailed characterization revealed that the special structure for some CdS pyramid structures dispersed in the MoS_2_ microsphere structures and surrounded by MoS_2_ nanosheets led to the photogenerated electrons migrating from the conduction band of different faces of the CdS pyramid to the conduction band of different MoS_2_ nanosheets while photogenerated holes remained in the CdS pyramid structures, which greatly promoted the separation of photogenerated electrons and holes, improving the photoactivity of the CdS/MoS_2_ catalyst. The catalyst also exhibited perfect stability, and the photoactivity displayed no significant degradation during continuous hydrogen production over nearly 70 h.

## Abstract 2

Schematic diagram of the photogenerated carrier migration between CdS and MoS_2_.

## Graphical abstract

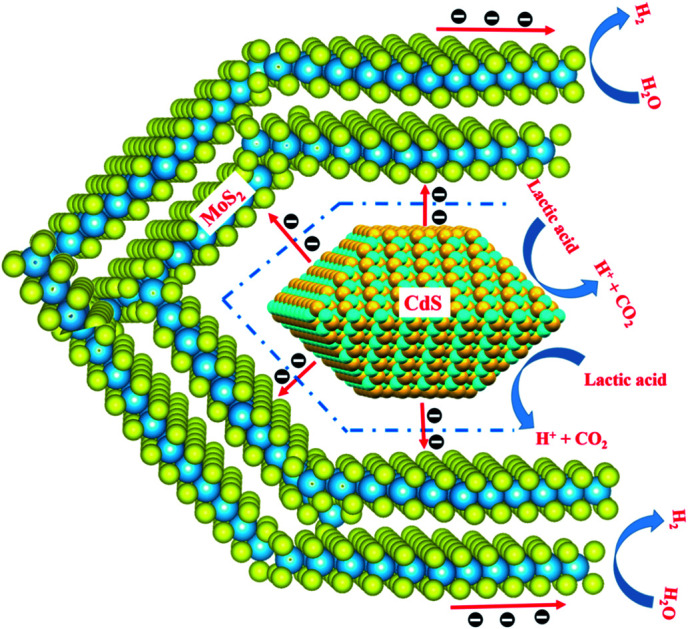

## 1. Introduction

Hydrogen is a type of clean and nonpolluting renewable energy that has attracted great interest and has the potential to solve the global energy crisis and reduce environmental pollution. An ideal approach for preparing hydrogen using solar energy to generate photocatalytic water splitting could convert solar energy to clean and storable hydrogen energy. The key issue to achieve hydrogen production from solar photocatalytic water splitting is to develop semiconductor catalysts with high activity and stability.^1–8^ Among many different kinds of photocatalysts, sulfide photocatalysts have more matching band gaps and higher visible light absorption efficiencies for hydrogen production, which have attracted much attention and have shown potential in industrial applications.^9–17^ CdS, in particular, is the most widely studied sulfide photocatalyst because of its small band gap (approximately 2.3 eV).^14^ Moreover, CdS loaded by some noble metals, including Pt, Pd, Ru and so on, showed a much higher photogenerated charge efficiency, which greatly improved the CdS photocatalytic hydrogen production activity.^18–20^ CdS loaded with 0.3 wt% Pt and 0.13 wt% PdS exhibited the best photocatalytic hydrogen production activity with an apparent quantum yield of 64.8% at 420 nm.^18,19^ However, considering the cost, some inexpensive metals and compounds as cocatalysts have attracted increasing interest.^21–25^ Investigations have indicated that MoS_2_-loaded CdS photocatalysts can also realize high photocatalytic activity.

Many highly active MoS_2_-loaded CdS photocatalysts have been reported in the literature.^26–43^ Zhao *et al.* reported a platinum-free 1D/2D CdS/MoS_2_ photocatalyst, and their experimental results showed that the highest hydrogen production rate of 1.79 mmol g^−1^ h^−1^ was obtained when the reaction ratio of CdS to MoS_2_ was 0.3.^41^ Yin *et al.* reported noble-metal-free CdS@MoS_2_ core–shell nanoheterostructures. 6 wt% MoS_2_-loaded CdS exhibited the best photocatalytic H_2_ evolution performance thus far, with a rate of 62.55 mmol g^−1^ h^−1^.^34^ Jiang *et al.* reported a berry-shaped (b)-CdS/MoS_2_ photocatalyst, and 1 wt% MoS_2_-loaded CdS exhibited the best photocatalytic H_2_ evolution performance thus far, with a rate of 63.59 mmol g^−1^ h^−1^.^40^ Reddy *et al.* successfully synthesized a few-layered black phosphorus/MoS_2_ nanohybrid as a promising cocatalyst, 8 wt% of which loaded on CdS nanorods showed a much higher hydrogen production rate of 183.24 mmol g^−1^ h^−1^.^35^ Ultrasmall cobalt nanocrystals embedded in 2D-MoS_2_ nanosheets as efficient cocatalysts for solar-driven hydrogen production were reported by Lee *et al.* Thus, a Co–MoS_2_ cocatalyst loaded on CdS nanorods showed a very high H_2_ production rate (275 mmol g^−1^ h^−1^) when the mass fraction of MoS_2_ loaded with 1 wt% Co was 6%, which was the most active MoS_2_-loaded CdS photocatalyst for hydrogen production reported in the literature.^33^

Through analysis of the abovementioned literature, only a small amount of MoS_2_ loaded on CdS exhibited a high photocatalytic activity for hydrogen production. Here, we report a kind of MoS_2_/CdS photocatalyst with flower-like MoS_2_ microspheres compounded with irregular CdS pyramids. The special structures led to the CdS/MoS_2_ photocatalyst exhibiting a superior separation efficiency for photogenerated electrons and holes. When the molar ratio of MoS_2_ to CdS was 1 : 1, the MoS_2_/CdS photocatalyst showed the highest hydrogen production rate of 394 mmol g^−1^ h^−1^, and the apparent quantum yield reached 64.8% at 420 nm. To our knowledge, this value is the highest efficiency ever reported for MoS_2_-modified CdS photocatalysts.

## 2. Experimental

### 2.1. Synthesis

All chemicals were of analytical grade and were used as received without any further purification. Cadmium acetate (Cd(CH_3_COO)_2_·2H_2_O), ammonium molybdate ((NH_4_)_6_Mo_7_O_24_·4H_2_O), thiourea (CH_4_N_2_S), and ethanol (C_2_H_6_O) were purchased from Sino-pharm Chemical Reagent of China.

Five proper amounts of ammonium molybdate (the molar of 0.01, 0.0071, 0.005, 0.0042 and 0.0033 mol) were dissolved in deionized water (70 mL) in Teflon tubes (100 mL), and then, appropriate amounts of Cd(CH_3_COO)_2_·2H_2_O (the molar ratios of Cd to Mo were 0.5, 0.7, 1, 1.2 and 1.5) and excessive thiourea were added to the solution, with stirring for several minutes. The Teflon tubes were transferred to autoclaves, which were sealed and heated at 200 °C for 24 h. The resulting precipitates collected by centrifugal separation were washed with deionized water and absolute ethanol several times and then dried under vacuum for 24 h at 80 °C. The catalysts prepared by this method were denoted as CdS/MoS_2_-*i* (molar ratios of Cd to Mo, *i* = 0.5, 0.7, 1, 1.2 and 1.5). “CdS/MoS_2_” was “CdS/MoS_2_-1” in “3. Results and discussion”.

Pure CdS and MoS_2_ were also prepared using the same process described above.

Based on CdS/MoS_2_-1, a Pt-loaded CdS/MoS_2_-1 catalyst was prepared by an *in situ* photoreduction method using a Xe lamp (300 W) equipped with a 420 nm cutoff filter, as follows: a certain amount of CdS/MoS_2_-1 was added to a 200 mL aqueous solution containing 10 vol% lactic acid and 0.25 M Na_2_SO_3_/0.35 M Na_2_S. A certain amount of chloroplatinic acid (2 wt% of the CdS/MoS_2_-1 quality) was added to the two aqueous solutions. Then, the Pt-loaded CdS/MoS_2_-1 catalyst was obtained after the aqueous solution was photoreduced for 1 h, denoted as Pt/CdS/MoS_2_-1.

Pure CdS and MoS_2_ were mechanically mixed, and then a Pt-loaded catalyst was prepared by the same method, denoted as Pt/CdS + MoS_2_.

### 2.2. Characterization

The X-ray diffraction (XRD) patterns of the as-prepared photocatalysts were obtained on a PANalytical X'pert MPD Pro X-ray diffractometer equipped using Cu-Kα irradiation. Scanning electron microscopy (SEM) images were obtained using a JSM-7800F-type field emission scanning electron microscope. X-ray photoelectron spectroscopy (XPS) measurements were obtained on an Axis Ultra Kratos (UK) multifunctional spectrometer with monochromatic Al Kα radiation. The ultraviolet visible (UV-vis) absorption spectra were measured on a HITACHI U-4100 spectrophotometer. Fluorescence spectroscopy was performed using a PTI QM-4 fluorescence spectrophotometer.

### 2.3. Photocatalytic hydrogen production

Photocatalytic reactions for hydrogen production were performed in a side irradiation Pyrex cell. A total of 0.01 g of catalyst powder (CdS/MoS_2_-*i*, Pt/CdS/MoS_2_–I or Pt/CdS + MoS_2_) was added to an aqueous solution (200 mL) containing 10 vol% lactate acid or 0.35 M Na_2_S/0.25 M Na_2_SO_3_ as electron donors. Nitrogen was purged in the cell to remove oxygen before irradiation. The solution was irradiated by visible light through a Xe lamp (300 W) equipped with a 420 nm cutoff filter. The amount of hydrogen was determined using TCD gas chromatography (NaX zeolite column, TCD detector, N_2_ as carrier gas). The apparent quantum yield (AQY) was calculated according to the equation reported in the literature.^11^

## 3. Results and discussion

**Fig. 1:**
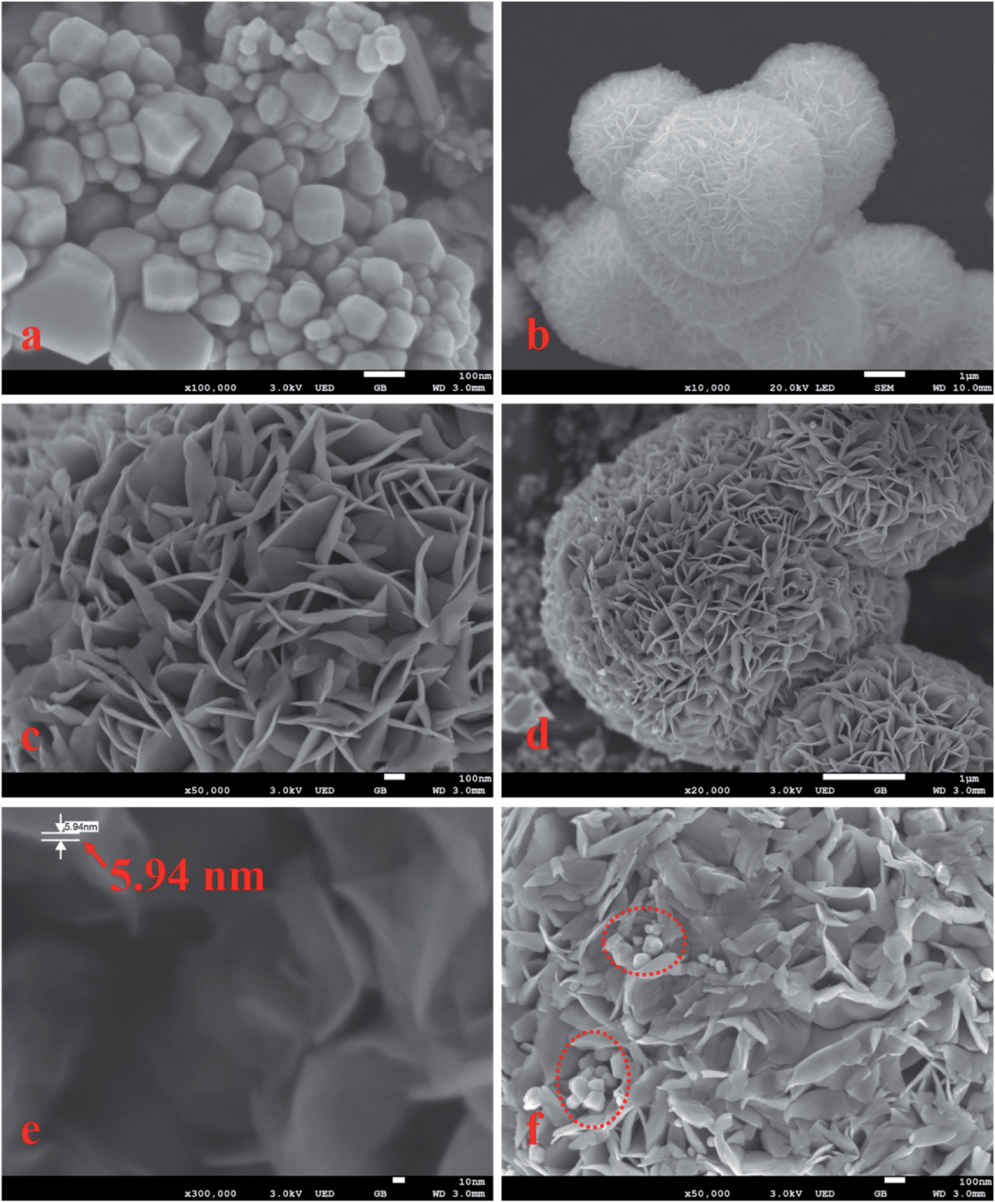
SEM images of CdS/MoS_2_ (a–f).

Fig. 1 shows SEM images of the samples. As shown in the images (Fig. 1a and b), the morphology of the samples presented differently sized irregular pyramid structures with particle sizes of 10–300 nm (Fig. 1a) and flower-like microspheres with particle sizes of 4–7 μm (Fig. 1b). The flower-like microsphere structures were self-assembled by a plurality of nanosheets with a thickness of 5.94 nm (Fig. 1c–e). Some irregular pyramid structures were surrounded by nanosheets with microsphere structures, and different surfaces of irregular pyramid structures directly contacted nanosheets, as shown in Fig. 1f. The irregular pyramid structures and flower-like microspheres were presumed to be CdS and MoS_2_, and in addition, some CdS pyramid structures were dispersed in the MoS_2_ microsphere structures.

**Fig. 2:**
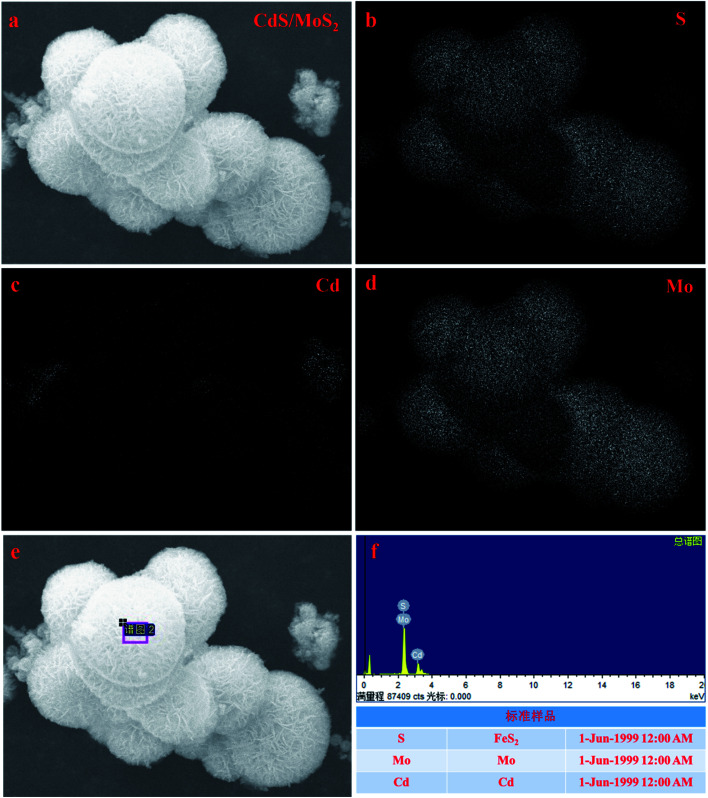
SEM image of CdS/MoS_2_ (a) and SEM mapping images of S, Cd and Mo (b–f).

**Fig. 3:**
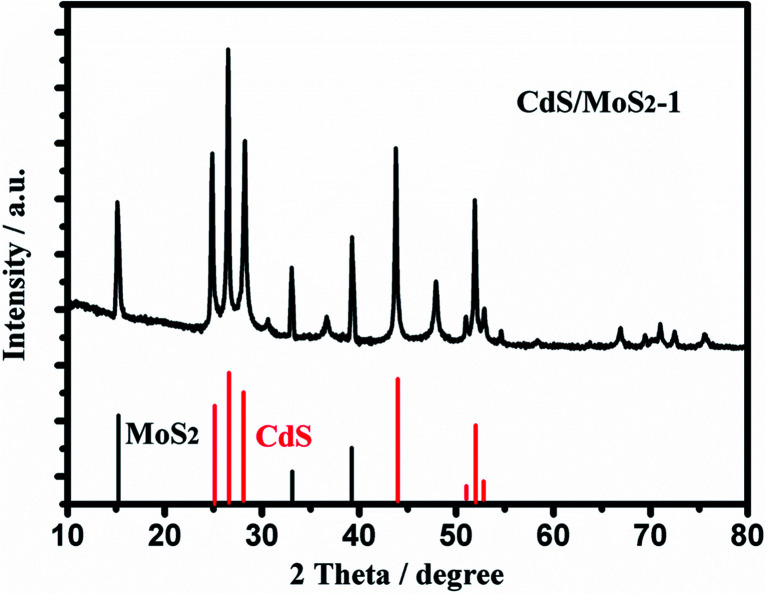
XRD patterns of CdS/MoS_2_.

To further prove the above inferred conclusion, SEM mapping of the CdS/MoS_2_ catalyst was performed, as shown in Fig. 2. As seen, S was distributed throughout the entire catalyst region (Fig. 2b), demonstrating that Mo and Cd were in the form of MoS_2_ and CdS in the catalyst. The distribution of Mo (Fig. 2d) was more intensive in the distribution region of the microsphere structures (Fig. 2a), illustrating that MoS_2_ formed microsphere structures in the catalyst. The content of Cd was very low, as shown in Fig. 2c, and was distributed in the MoS_2_ microsphere structures (Fig. 3c). Some box samples in Fig. 2c were used for element analysis, and the results are shown in Table 1. It can be seen from the table that the microsphere structure was MoS_2_, and a small amount of CdS pyramid structures was dispersed in the MoS_2_ microsphere structure.

**Table 1:** Local element composition of CdS/MoS_2_

Element	Element concentration	Intensity correction	Weight percent	Weight percent sigma	Atom percent
S K	14.03	1.0538	29.61	0.34	57.93
Mo L	12.16	0.9325	29.00	0.60	18.96
Cd L	12.55	0.6741	41.39	0.42	23.10
Total			100.00		

Fig. 3 shows the XRD patterns of CdS/MoS_2_-1. As shown from the patterns, the diffraction peaks of the hexagonal phase CdS (JCPDF no. 41-1049) appeared at 25°, 26°, 28°, 51°, 52° and 53°, and the diffraction peaks of cubic phase CdS (JCPDF no. 45-0647) appeared at 26°, 31°, 37°, 44°, 48° and 52°. The diffraction peaks of hexagonal phase MoS_2_ (JCPDF no. 75-1539) appeared at 15.1°, 34.2° and 39.5°. Compared with pure CdS and MoS_2_, the diffraction peaks of CdS/MoS_2_-1 showed no movement with the addition of MoS_2_ or CdS, which revealed that MoS_2_ and CdS only contacted the surface instead of being absorbed into each other's lattice.

**Fig. 4:**
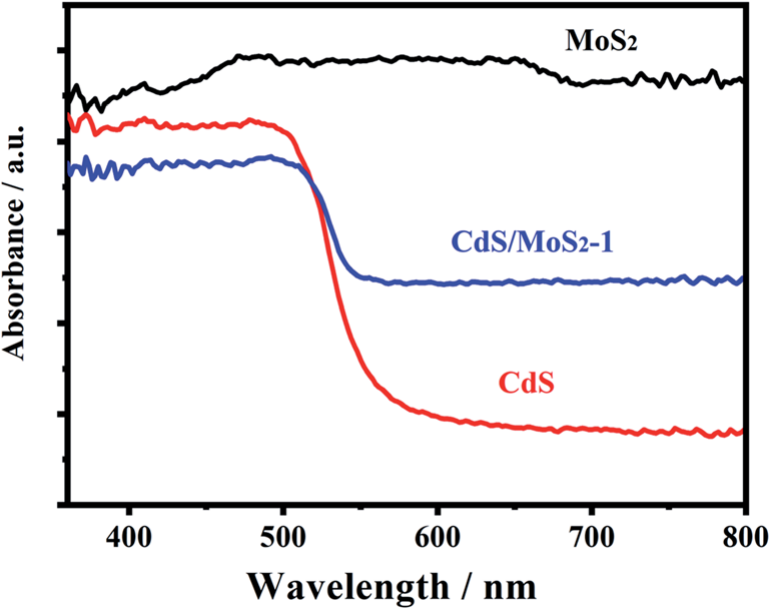
Uv-vis absorption spectra of pure CdS, pure MoS_2_ and CdS/MoS_2_-1.

Fig. 4 shows the UV-vis absorption spectra of pure CdS, pure MoS_2_ and CdS/MoS_2_-1. Pure MoS_2_ had absorption over the whole wavelength range of 400–800 nm, while the absorption edge of pure CdS was located at 536 nm. The band gaps of pristine CdS and MoS_2_ were estimated to be 2.31 eV and 1.32 eV, respectively, by the Kubelka–Munk function.^11^ CdS/MoS_2_-1 showed an apparent enhancement of the visible light absorption from 536 to 800 nm, which was attributed to the absorption of MoS_2_ over the entire wavelength range of 400–800 nm. It was revealed that the narrow band gap of MoS_2_ could improve the visible absorption of CdS/MoS_2_ heterojunctions. The enhanced light absorption of the catalysts was expected to favor the formation of more photogenerated electrons available for photocatalytic hydrogen production.

**Fig. 5:**
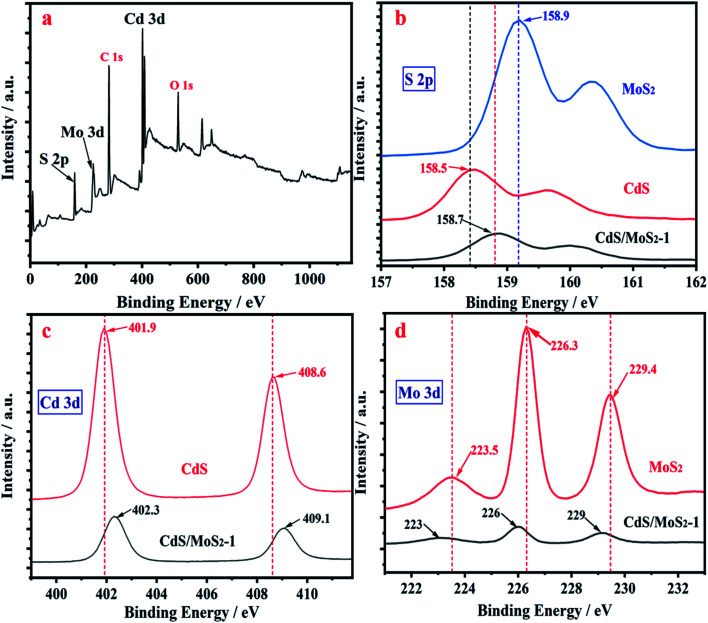
XPS spectra of CdS/MoS_2_ (a); high-resolution XPS spectra of S 2p (b); high-resolution XPS spectra of Cd 3d (c) and of Mo 3d (d) for pure CdS, MoS_2_ and CdS/MoS_2_.

The peaks of Cd, Mo and S electrons in the XPS survey scan spectra of CdS/MoS_2_ shown in Fig. 5a were identified for the samples, confirming the successful synthesis of CdS/MoS_2_. Fig. 5b–d show high-resolution XPS spectra of S 2p, Cd 3d and Mo 3d for pure CdS, MoS_2_ and CdS/MoS_2_, respectively. Compared to pure CdS and MoS_2_, the absorption peaks of Cd 3d and S 2p for CdS/MoS_2_ were redshifted by 0.4 eV and 0.2 eV, while the absorption peaks of Mo 3d and S 2p for CdS/MoS_2_ were blueshifted by 0.3 eV and 0.2 eV, respectively, which were mainly derived from the electronic interaction between CdS and MoS_2_. The redshift and blueshift revealed that the density of electrons in CdS decreased and that in MoS_2_ increased, resulting in an increase and decrease in the binding energy for CdS and MoS_2_, respectively; thus, it was inferred that photoelectrons transferred from CdS to MoS_2_, which achieved efficient separation of photogenerated charges between CdS and MoS_2_, improving the photocatalytic activity for hydrogen production.

**Fig. 6:**
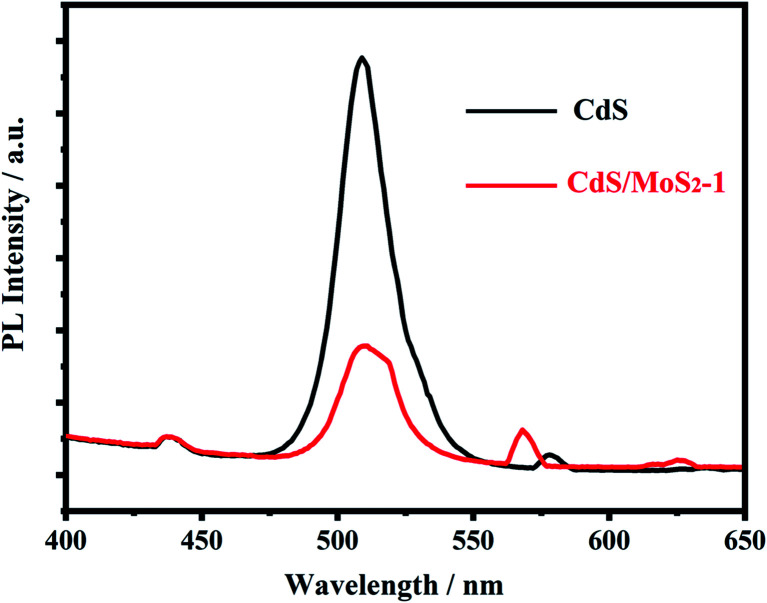
Photoluminescence spectra of pure CdS and CdS/MoS_2_, excitation 370 nm.

The photoluminescence spectra of pure CdS and CdS/MoS_2_ were also measured and are shown in Fig. 6. The spectra present emission peaks at 475–550 nm, which were related to the recombination process of electrons and holes in the semiconductor. Compared with pure CdS, the CdS/MoS_2_ peak showed a much lower intensity, revealing that CdS/MoS_2_ exhibited a superior efficiency of separation for photogenerated electrons and holes, which improved the photocatalytic activity for hydrogen production.

**Fig. 7:**
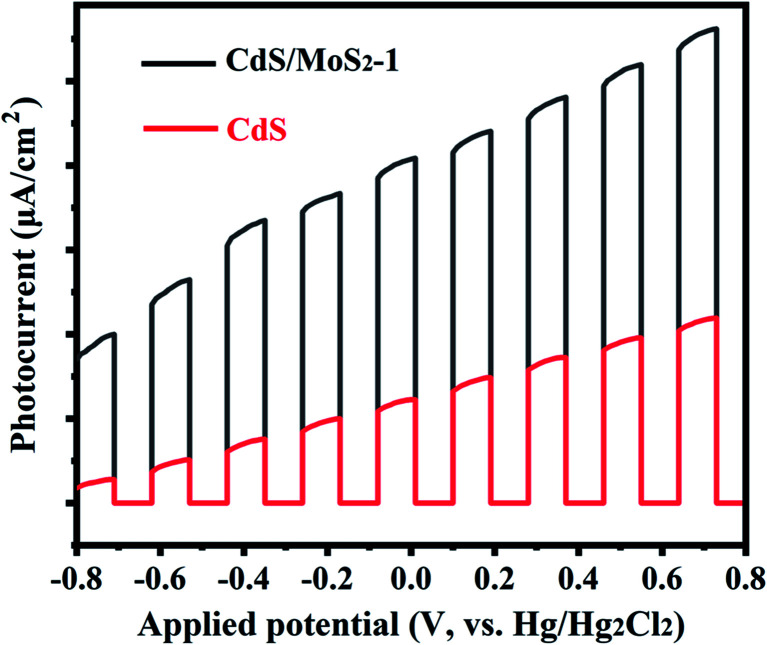
The current–potential characteristics of as-prepared pure CdS and CdS/MoS_2_-1. Test condition: A 300 W Xe lamp coupled with an AM 1.5 filter was used as the light source; photocurrent density was measured in the 0.5 M Na_2_SO_3_ aqueous solution as the electrolyte; Hg/Hg_2_Cl_2_ was as the reference electrode.

Photocurrent experiments were also performed to further illustrate the charge transfer behavior for CdS/MoS_2_-1 and pure CdS, as shown in Fig. 7. The photoelectrodes for photoelectrochemical test samples were prepared by electrophoretic deposition (EPD) onto fluorine-doped tin oxide (FTO)-coated glass substrates. As shown in Fig. 7, the photocurrent density of the samples increased gradually with increasing applied potential. Compared with pure CdS, CdS/MoS_2_-1 exhibited an extremely higher photocurrent density, indicating that CdS/MoS_2_-1 showed more efficient charge separation than pure CdS, which improved the photocatalytic activity for hydrogen production.

**Fig. 8:**
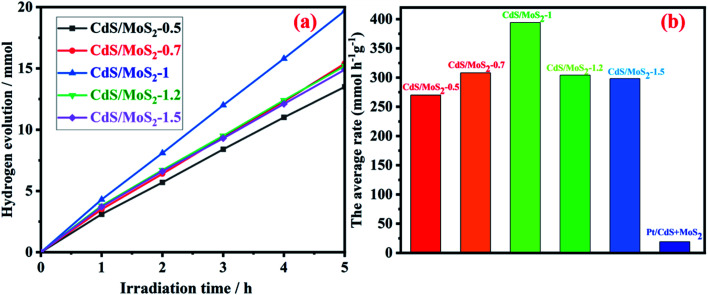
Photocatalytic hydrogen production activities of CdS/MoS_2_-*i* (the molar ratio of Cd to Mo, *i* = 0.5, 0.7, 1, 1.2 and 1.5) and Pt/CdS + MoS_2_. Reaction condition: 0.01 g of catalyst; 200 mL of aqueous solution containing 180 mL deionized water and 20 mL lactate acid; 300 W Xe lamp equipped with a cutoff filter (*λ* > 420 nm).

The amount and average rate of photocatalytic hydrogen production during 5 h irradiation for CdS/MoS_2_-*i* (molar ratios of Cd to Mo, *i* = 0.5, 0.7, 1, 1.2 and 1.5) are shown in Fig. 8. With increasing molar ratio, the amount and average rate of hydrogen production increased gradually and then reached maximum values of 19.7 mmol and 394 mmol h^−1^ g^−1^, respectively, for CdS/MoS_2_-1. The amount and average rate decreased as the molar ratio continued to increase. The average rates of photocatalytic hydrogen production for mechanically mixed CdS and MoS_2_ were also measured. The experimental results of hydrogen production showed a low average rate of 19 mmol h^−1^ g^−1^ for mechanically mixed CdS- and MoS_2-_loaded Pt. It was revealed that the interface between CdS and MoS_2_ in CdS/MoS_2_ contributed to the separation of photogenerated electrons and holes.

**Fig. 9:**
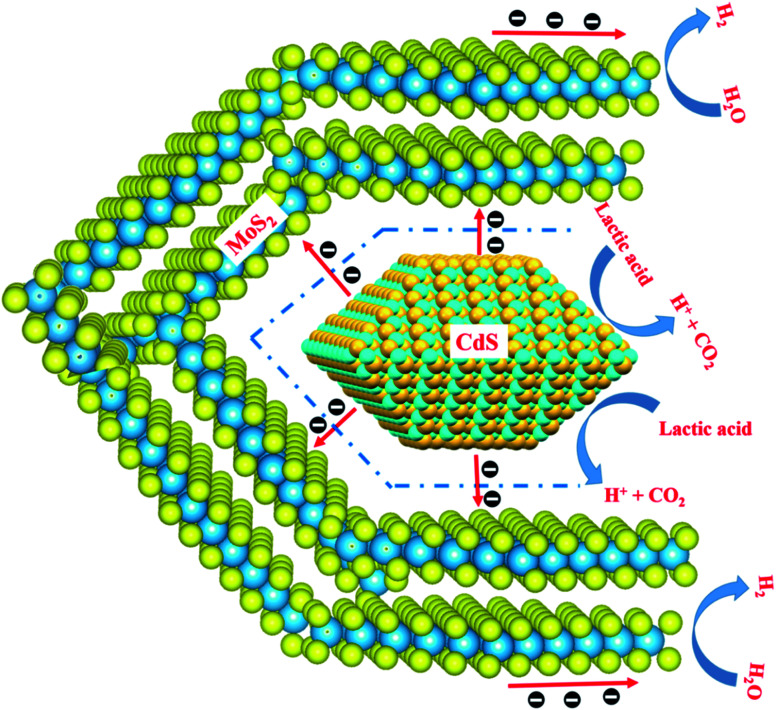
Schematic diagram of the photogenerated carriers migration between CdS and MoS_2_.

**Fig. 10:**
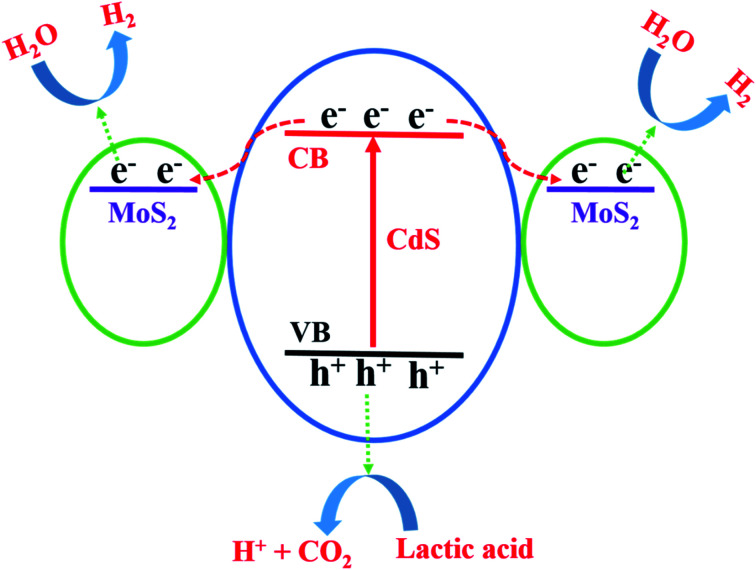
Migration mechanism of the photogenerated carriers in CdS/MoS_2_.

Through all of the characterization analyses, it was known that the CdS/MoS_2_ photocatalyst exhibited a superior efficiency of separation for photogenerated electrons and holes, which was attributed to the special structure of some CdS pyramid structures dispersed in the MoS_2_ microsphere structures and surrounded by MoS_2_ nanosheets. The different faces of the CdS pyramid structures contacted the surface of the MoS_2_ nanosheets to form heterojunctions. Through the heterojunctions, photogenerated electrons could be transferred from the different faces of CdS pyramid structures to different MoS_2_ nanosheets, while photogenerated holes remained in CdS pyramid structures. Many highly active dangling bonds existed at the edge of the nanosheets, which could form chemical bonds with H. The ability to bond with H provided the best medium for the convenient adsorption and desorption of H on the surface of the photocatalyst, accelerating the photochemical reaction rate of the photocatalyst interface. Therefore, the photogenerated electrons could continue to migrate to the edge of the MoS_2_ nanosheets with highly active dangling bonds and then react with H^+^ to form H_2_, while the photogenerated holes were consumed by the lactic acid on the surface of the CdS pyramid structures, as shown in Fig. 9. Based on the above analysis, it was revealed that photogenerated electrons migrated from the conduction band of different faces of the CdS pyramid to the conduction band of different MoS_2_ nanosheets, while photogenerated holes remained in the CdS pyramid structures, which greatly promoted the separation of photogenerated electrons and holes, improving the photoactivity of the CdS/MoS_2_ catalyst, as shown in Fig. 10.

**Fig. 11:**
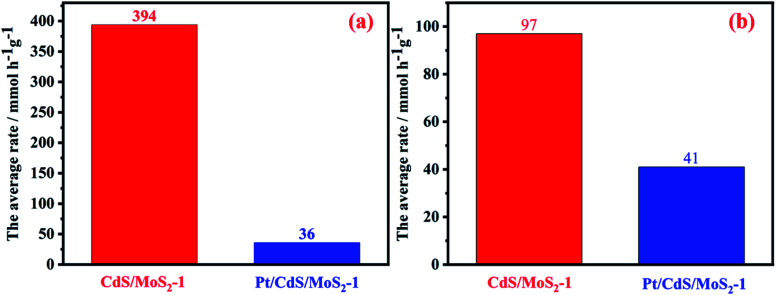
The average rates of photocatalytic hydrogen production for CdS/MoS_2_-1 and Pt loaded CdS/MoS_2_-1. Reaction conditions (a): 0.01 g of catalyst; 200 mL of lactate acid aqueous solution (lactate acid of 10 vol%); 300 W Xe lamp equipped with a cutoff filter (*λ* > 420 nm); reaction conditions (b): the same but substituting 0.25 M Na_2_SO_3_/0.35 M Na_2_S solution for the lactate acid solution.

It is known that the photogenerated electrons could be transferred from the CdS to MoS_2_ or Pt, while photogenerated holes remained in CdS, resulting in a superior efficiency of separation for photogenerated electrons and holes in the CdS catalyst, and improving the photocatalytic hydrogen production efficiency for CdS catalyst. The photoactivity of CdS was greatly improved by coloaded Pt, mainly because Pt could capture photogenerated electrons from CdS, further promoting the separation of photogenerated carriers. To provide additional evidence for the migration path of the photogenerated electrons between CdS and MoS_2_ in the CdS/MoS_2_ catalyst, the hydrogen production activity for Pt-loaded CdS/MoS_2_-1 was studied, as shown in Fig. 11a. The results showed that the average rate of photocatalytic hydrogen production for CdS/MoS_2_-1 was much higher than that of Pt-loaded CdS/MoS_2_. It is known that the Pt-loaded CdS catalyst exhibits excellent photoactivity in the sacrificial agent system of Na_2_SO_3_/Na_2_S. To eliminate this effect, the activity of hydrogen production for Pt-loaded CdS/MoS_2_-1 in a 0.25 M Na_2_SO_3_/0.35 M Na_2_S solution was studied, as shown in Fig. 11b. The results showed that the average rate of CdS/MoS_2_-1 was still higher than that of Pt-loaded CdS/MoS_2_-1. According to the above experimental results, photogenerated electrons migrated from the conduction band of CdS to the conduction band of MoS_2_. Pt could capture photogenerated electrons of CdS, leading to a decrease in photogenerated electron migration to MoS_2_ conduction. This, in turn, resulted in a decrease in the hydrogen production activity for CdS/MoS_2_. This finding was consistent with the experimental results.

**Fig. 12:**
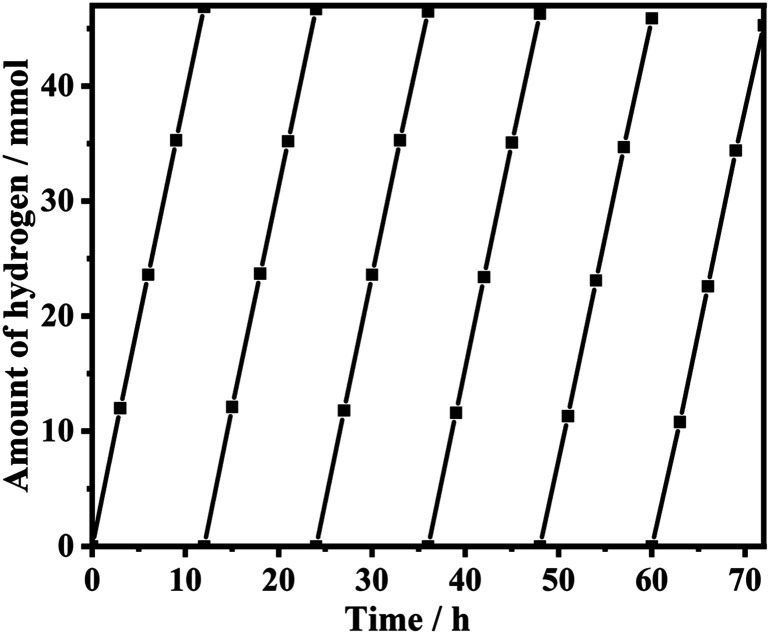
Long-term photocatalytic hydrogen evolution measurement over CdS/MoS_2_-1. Reaction conditions: 0.01 g of catalyst; 200 mL of lactate acid aqueous solution (lactate acid of 10 vol%); 300 W Xe lamp equipped with a cutoff filter (*λ* > 420 nm).

The stability of CdS/MoS_2_-1 in a lactic acid solution was studied, as shown in Fig. 12. CdS/MoS_2_-1 exhibited perfect stability, the photoactivity displayed no significant degradation during continuous hydrogen production over nearly 70 h, and the photoactivity of CdS/MoS_2_-1 was reduced by only 1.6 mmol after 72 h of photocatalytic reaction.

## 4. Conclusions

In summary, an irregular CdS pyramid/flower-like MoS_2_ microsphere composite photocatalyst was successfully synthesized, achieving a hydrogen evolution rate of 394 mmol g^−1^ h^−1^ with an extremely high apparent quantum yield (AQY = 64.8%) at 420 nm. To our knowledge, this value is the highest efficiency ever reported for MoS_2_-modified CdS photocatalysts. Because of the special structure of some CdS pyramid structures dispersed in the MoS_2_ microsphere structures and surrounded by MoS_2_ nanosheets, the photogenerated electrons migrated from the conduction band of different faces of the CdS pyramid to the conduction band of different MoS_2_ nanosheets. Meanwhile, photogenerated holes remained in the CdS pyramid structures, which greatly promoted the separation of photogenerated electrons and holes, improving the photoactivity of the CdS/MoS_2_ catalyst. The catalyst also exhibited perfect stability, and the photoactivity displayed no significant degradation during continuous hydrogen production over nearly 70 h. This research has some guiding significance for promoting the study of low-cost and efficient photocatalytic hydrogen production.

## Conflicts of interest

There are no conflicts to declare.

## Supplementary Material
